# Recent Progress on Ex Situ Remediation Technology and Resource Utilization for Heavy Metal Contaminated Sediment

**DOI:** 10.3390/toxics11030207

**Published:** 2023-02-23

**Authors:** Qinqin Xu, Boran Wu

**Affiliations:** State Key Laboratory of Pollution Control and Resource Reuse, College of Environmental Science and Engineering, Tongji University, 1239 Siping Road, Shanghai 200092, China

**Keywords:** sediment, heavy metal, ex situ remediation, resource utilization

## Abstract

Sediment is an important part of aquatic systems, which plays a vital role in transporting and storing metals. Due to its abundance, persistence, and environmental toxicity, heavy metal pollution has always been one of the hot spots in the world. In this article, the state-of-art ex situ remediation technology for metal-contaminated sediments is elaborated, including sediment washing, electrokinetic remediation (EKR), chemical extraction, biological treatment, as well as encapsulating pollutants by adding some stabilized/solidified materials. Furthermore, the progress of sustainable resource utilization methods, such as ecosystem restoration, construction materials (e.g., materials fill materials, partition blocks, and paving blocks), and agriculture use are reviewed in detail. Finally, the pros and cons of each technique are summarized. This information will provide the scientific basis for selecting the appropriate remediation technology in a particular scenario.

## 1. Introduction

Heavy metals are natural components of the Earth’s crust. In rocks, they occur as minerals in different chemical forms, such as sulfides and oxides [1]. With rapid industrialization, various anthropogenic activities (e.g., treated and recycled minerals, product manufacturing, burning fuels (particularly coal) and developing new oil fields, modern agricultural practices, and waste disposals contribute to the release of heavy metals [2]. Once metals (such as Hg, Cd and Pb) enter into the atmosphere, they can be transported over long distances by air currents, leading to the dramatic increase of heavy metal content in remote areas [3,4,5,6]. In addition, through drainage, atmospheric bulk deposition, and soil erosion, heavy metals are prone to enter into water bodies and disturb the aquatic ecosystem [7]. Due to their persistence, metals are bioaccumulative and bioamplifying in predatory animals (e.g., fish) that can be used as food for humans. Exposure to heavy metals above maximum acceptable levels can cause a range of damage, disease, and even cancers to the body by disrupting the tumor suppressor gene expression, damaging repair processes and DNA, inducing oxidative stress and cell death process [8]. Hence, heavy metal pollution has always been one of the hot spots in the world.

As heavy metals are transported into the water, most fractions (>90%) are trapped in sediments by adsorption, precipitation, complexation, and chelation reactions [9]. The toxicity and fluidity of metals depend on their distribution and morphology. According to Tessier’s sequential extraction, heavy metals in sediment are divided into five fractions: extractable and exchangeable, carbonate bound, Fe-Mn oxides bound, organic matter bound, and residual metal, in which organic fraction and residual fraction are less available for biota [10,11]. Over the past period from 1970 to 2017, metals of Cr, Cu, Ni, Cd, and Mn showed an increasing trend in global river and lake water, and the cancer risks related to Cr exceeded the hazardous level [12]. In the same time period, the contents of Cr, Ni, Cd, Mn, and Co in global river sediments and Pb, Hg, Cr, and Mn in lake sediments exhibited an increasing trend; average metal concentrations are generally higher in Europe and North America than in Africa, Asia, and South America [13]. Thus, regional pollution control measures and integrated environmental and ecological remediation technologies are highly necessary for heavy metal polluted rivers and lakes.

**Figure 1:**
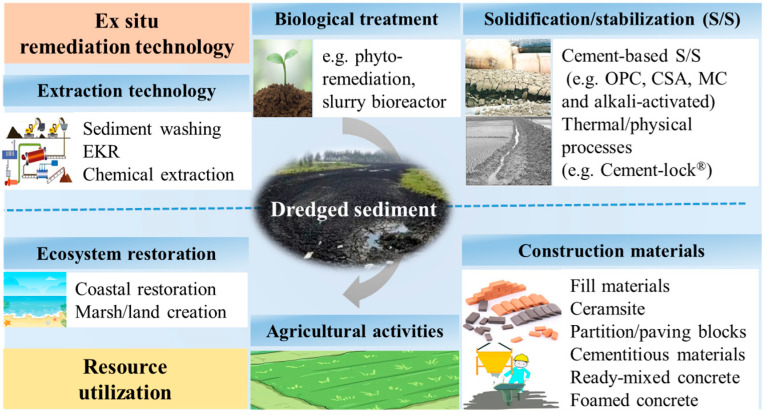
Ex situ remediation technology and resource utilization for heavy metal contaminated sediment.

Traditionally, remediation of metal-contaminated sediments has been achieved through in situ and ex situ treatment [14,15]. In situ treatments are considered to be a less invasive treatment method but their implementation is site specific, challenging, and expensive due to multiple factors affecting specific fixation mechanisms and geochemistry [16]. Ex situ remediation separates contaminated sediment from the river bed and reduces the release of pollutants to overlying water, which can fundamentally remove pollutants and is more appropriate for heavily contaminated sediments [10]. Ex situ processes remove metals in dredged sediment via sediment washing, EKR and chemical extraction, biological treatment, or encapsulating pollutants chemically and physically by adding some stabilized/solidified materials (such as cement, fly ash, lime, and AAMs) (Figure 1) [14,17]. Although it is long-playing and needs an offsite location to treat dredges sediment, the treated sediment can be recycled in a variety of ways (such as ecosystem restoration, making construction materials (e.g., materials fill materials, partition blocks, and paving blocks) and agriculture use) to achieve sustainable development of the resource (Figure 1) [14,18,19,20]. Indeed, most of the available papers reviewed remediation technologies (including in situ and ex situ) for heavy metal contaminated sediment in recent years but there are few comprehensive introductions of the progress on ex situ remediation technologies and resource utilization approaches. In this review article, the objectives are to: (1) elaborate the state-of-art ex situ remediation of metal-contaminated sediments; (2) review the recent sustainable resource utilization methods; and (3) summarize pros and cons of each technique and prospect possible future research directions.

## 2. Ex Situ Remediation Technology

### 2.1. Extraction Technology

#### 2.1.1. Sediment Washing

Sediment washing extracts metals from dredged sediment to the washing solution through high-pressure water jets or adding some chemical washing agents (such as inorganic acid, chelators, and surfactants) (Table 1) [10]. As for washing agents, inorganic acid (HCl, HNO_3_, and H_2_SO_4_) is recognized as effective in extract metals [21,22]. The main mechanism for HCl is the enhanced desorption by low pH, the dissolution of discrete metal compounds, and the specific composition containing metals (such as Fe-Mn oxides), whereas too many chloride ions may result in the formation of a protective layer around solid particles restricting metal removal [21]. HNO_3_ may have a higher capacity of destroying metal-organic complexes than HCl due to its strong oxidation [23]. Moreover, the additives of chelators (e.g., EDDS, EDTA, organic acid (AA and CA)) have been extensively studied and applied to increase the solubility of heavy metals [7]. Particularly, EDTA is the most efficient extractant because it can form very stable complexes with metals [24] but a significant part of metal-EDTA complexes remain in the soil/sediment due to adsorption on the mineral surface [25]. In addition, surfactants (e.g., rhamnolipid, HS) can enhance the solubilization diffusion and desorption of metal contaminants in contaminated sediments [26,27]; the mechanism of heavy metal removal is that of biosurfactant molecules complex with metals and transfer to the solution phase in the form of aggregation into micelles (spherical bilayer with a diameter less than 50 nm) [28]. Among them, biosurfactants are cost-effective and eco-friendly washing chemicals with low toxicity, biodegradability, and surface activity [26,27].

#### 2.1.2. Electrokinetic Remediation (EKR)

EKR can be applied both in situ and ex situ, which involves inserting electrodes into a contaminated medium and applying an electric field to move the contaminant towards the electrodes [38]. This is an extremely complex process including electrochemical reactions (e.g., water electrolysis on the surfaces of the electrodes) and geochemical behaviors (e.g., adsorption-desorption reaction, precipitation dissolution reaction, and crystalline reaction in the solution or the porous medium) [39]. The main transport mechanisms of contaminants are electromigration, electroosmosis, and electrophoresis [40]. Electrolysis of water molecules is an important reaction at an electrode that produces hydrogen (H^+^) (H_2_O → 2H^+^ + 1/2 O_2_ (gas) + 2e^−^) and hydroxide ions (OH^−^) (2H_2_O + 2e^−^ → 2OH^−^ + H_2_ (gas)), which not only polarizes the electrodes, but also changes the pH of sediment with possible repercussions on its chemistry [41]. On the other hand, the precipitation of heavy metals caused by OH^−^ greatly limits the removal efficiency of heavy metals, despite electrolytes containing suitable complexants, chelators, surfactants, and other agents that help to overcome this problem [38,42]. However, the presence of Cl^−^ in the electrolyte can be oxidized to produce Cl_2_ gas, which can compete with water oxidation and reduce the formation of H^+^ at the anode, thus affecting the adsorption and desorption reactions of metal ions [43]. Similarly, the initial salinity of sediments (especially for marine sediments) can also lead to high levels of Cl_2_ gas production at the anode [44]. Besides, there are some other side effects appearing in EKR processes, such as thermal effects, crystallization effect, electrode corrosion reactions and so on [43]. In recent years, EKR coupled with PRB has been developed to improve efficiencies and achieve the degradation or recycling of pollutants migrating from the soil [45]. PRB embeds reactive materials that react with the contaminants to capture or degrade them. Commonly used filling materials are zeolite, active carbon, Fe based material, nanomaterials (e.g., nZVI), and some modified materials, which often achieve excellent performance and maintain high sustainability [43,46]. Compared with EKR alone, the heavy metal removal rate of EKR-PRB technology can reach up to 97% [43]. In terms of energy efficiencies, pulsed electric fields and self-powered technologies (e.g., solar-power and microbial fuel cells) have been widely studied (Table 2) [47,48,49].

#### 2.1.3. Chemical Extraction

Floatation is a possible technique for remediation of dredge sediment, which is obviously affected by the sediment properties (e.g., particle size), bubbles, and extraction agents [59,60,61]. In floatation, collectors are the most important reagents, which attach to mineral surfaces making them hydrophobic and promoting bubble attachment [62]. Transition metals (e.g., Cd, Cu, Pb, Zn, etc.) presenting in river sediments as sulfides can be selectively separated from suspensions by a collector-free floatation method [63]. If the metals are not originally in sulfides form, they can be chemically pretreated by hydroxylation and sulfidisation [62]. Removal efficiencies for most heavy metals in sediments can reach up to 80% in the floatation process [63] but the extractability of this method is lower than that of other remediation techniques [10].

Recently, microwave and ultrasonic-assisted heavy metal extractions have been emerging, which have the advantages of labor-less, time-saving, lower reagents usage, reduced contamination, and enhanced operator safety in comparison with conventional chemical extraction (Table 3) [64,65,66]. Ultrasonic irradiation can make solid suspended particles disperse, increase the available surface area for the reaction, and improve the ability of the extractant to leach metals [67]. Microwave energy utilizes permanent dipole rotation and unorganized movement of molecules to rapidly heat samples to shorten preparation time, which replaces the magnetic shaking and conventional heating in traditional extractions [68,69]. In addition, ILs have replaced organic solvents in extraction and microextraction processes, being an environmentally friendly regent [70]. ILs are a group of organic salts composed of different anions and organic cations, which is designable for task specific extraction of analytes from a variety of solvent media [70,71]. However, microwave and ultrasonic-assisted extractions are rarely applied by practical engineers.

### 2.2. Biological Treatment

Ex situ phytoremediation seems to have strong potential for treatment contaminated sediments. Contaminated dredged sediments are usually fine texture, and some are low nutrient content or high salinity [80], so pretreatments are necessary, such as mixing sediment with sand soil to improve its hydraulic properties, or covering a layer of compost on top of the sediment, or planting a mix of plants with different functions or adding other organisms (e.g., earthworms) to create an active ecosystem [81,82]. In tropical and subtropical regions, mangroves are typical ex situ phytoremediation plants, especially for a salt secreting species—*Avicennia* [81]. For instance, the root of *Acicennia marina* could accumulate Pb to 412 µg/g, Cu to 901 μg/g, and Zn to 1136 μg/g after remedying sediment for 7 months, respectively [83]; and Fe concentrations in roots and shoots of *Avicennia schaueriana* reached up to 85 μg/g and 100 μg/g after 8 months, respectively [84]. Beyond that, Ramie, pasture grass (*Lolium perenne*, *Medicago sativa*, *Festuca arundinacea*, *Cichorium intybus*), *Paspalum vaginatumand*, and *Tamarix gallica* all can be applied in metal remediation [82,85,86].

Bioleaching is the application of acidophilic microorganisms capable of Fe/S oxidative metabolism to promote metal dissolution in solid substrates, which is normally conducted as a sediment/soil slurry bioreactor [87]. The mechanism involves two aspects: one is that bacteria catalyze the oxidation of insoluble metal sulfides to soluble metal sulfates, and the other is that bacteria oxidizing elemental sulfur or reduced sulfur compounds to sulfuric acid, thus enhancing the solubility of the metal [88]. The main microbes are α-β-γ-Proteobacteria (*Acidiferrobacter*, *Acidiphilium*, *Acidithiobacillus*, *Ferrovum*), Actinobacteria (*Ferrimicrobium, Ferrithrix, Acidimicrobium*), Firmicutes (*Alicyclobacillus*, *Sulfobacillus*), Nitrospirae (*Leptospirillum*), as well as the archaea of Crenarchaeota (*Acidianus*, *Metallosphaera*, *Sulfolobus*, *Sulfurisphaera*) [87]. Although research on bioleaching has mostly focused on laboratorial scale, it is expected to be a promising environmental technology. For example, Chen et al. [89] established a continuous bioleaching process for contaminated sediment (from Ell Ren River) and founding the maximum solubilization for metals (Zn, Ni, Cu and Cr) could be achieved to 90%; the dominated bacterial community was acidophilic sulfur-oxidizing bacteria; Sabra et al. [90] utilized filamentous fungi and lithotrophic bacteria to leach metals from dredged sediments (from Deûle Canal, France); the bioleaching percentages varied from 72 to 93% for Cd, Cu, Mn, and Zn.

### 2.3. Solidification/Stabilization (S/S) Technology

S/S technology is used to immobilize contaminants both physically and chemically, which is evidently the most suitable for treatment of metal wastes [91]. Solidification is the process that converts the wastes of liquid, semisolid, or a powder into free-standing and monolithic masses with improved engineering mechanical properties, whereas stabilization is to reduce the mobility, solubility, and toxicity of contaminants through chemical interactions (such as adsorption, hydroxides, oxidation-reduction, etc.) [92]. After treatment of S/S, dredged sediment is convenient for handling and transporting to landfill sites or resource utilization as filling and supporting materials for engineering purposes [93,94]. According to the application of main stabilizing agents, S/S technology is mainly classified as a cement-based process and a thermal and physical process, in which cement-based process is most widely used, including the materials of ordinary Portland cement (OPC), calcium sulfoaluminate cement (CSA), magnesia-based cements (MC), and alkali-activated cement. Major research results in recent years are investigated and shown in Table 4. However, organic matter, salts, clay, and contaminants in the sediment can seriously affect the hydration of the cement, hindering its strength and growth, and preventing metal stabilization [95].

#### 2.3.1. Cement-Based Process

OPC is a hydraulic binder mainly comprised of alite, belite, aluminate, and ferrite [115]. It hydrates to produce C-S-H gel that contains the bulk of microporosity with high surface areas, which has a strong capacity for binding metals [17,116]. However, when metals are present, OPC hydration can be modified so that three-dimensional structures containing heavy metals can form up to 100–300 nanometers of thick coating around cement grains [17]. Metals that form hydroxides can affect hydration reactions depending on their solubility, that is, the insoluble hydroxides retard hydration reactions and the soluble hydroxides accelerate cement hydration [117]. Thus, the presence of heavy metal pollutants would increase the pore size of OPC and reduce its compressive strength [118].

When OPC blends with supplementary cementitious materials (e.g., fly ash, kiln dust, or silica fume), they would have pozzolanic reactions during hydration. Pozzolanic materials themselves are not cementitious, but when pozzolanic cement is hydrated, reactive oxides (e.g., SiO_2_, A1_2_O_3_, Fe_2_O_3_, etc.) react with calcium hydroxide to generate mainly calciumsificate-hydrate, calcium-aluminatehydrate, calcium-aluminate-silicate-hydrate, and calcium-alurninoferrohydrates phases [119]. Reaction products can infill the initial internal porosity of the cement paste, forming high density and high strength pore structure, and thus reducing pollutant mobility [115]. Besides, the hydration of lime-pozzolan cements (e.g., lime flyash and lime kiln dust) also have pozzolanic reactions, but lime processes are not as effective in the immobilization of heavy metals as cement-based systems [92,120].

CSA are traditionally composed of ye’elimite (major phase), belite, ferrite, ternesite, anhydrite (and/or gypsum), or mayenite [121]. The main hydration phase of CSA is ettringite, of which the columnar and channel sections of its crystal structure can act as a host for a number of metal ions, replacing the calcium, aluminium, hydroxide, and sulfate sites [116,122,123]. Moreover, its hydration products (especially ettringite) can imbibe large amounts of water and reduce internal humidity, thus inhibiting the corrosion of incoming metal [124]. Compared with OPC, CSA has advantages of low-carbon, low alkalinity, high early strength, good durability, and compatibility [123].

MC are now receiving attention due to its great performance for solidifying heavy metals, especially for magnesium potassium phosphate cement (MKPC) and magnesium silicate hydrate (M-S-H) cement [118,125,126,127,128]. MKPC mainly takes dead-burned MgO and phosphate as raw materials. The main hydration product is struvite (MgNH_4_PO_4_·6H_2_O) or MgKPO_4_·6H_2_O that can physically fix the heavy metals, and the residual phosphate has a chemical stabilization effect on heavy metals [129]. M-S-H cements are based around the formation of a M-S-H phase that aluminum can be incorporated into, existing in both tetrahedral and octahedral sites [127]. Besides, the curing effect of the M-S-H cement on heavy metals is mainly physical encapsulation and chemisorption of hydration products [128]. In comparison with OPC, magnesia-based cements have a better curing effect on heavy metals and the solidified body is much smaller [128].

Alkali-activated materials (AAMs) are prepared by the reaction of an aluminosilicate precursor (e.g., slag, fly ash, hybrid binders) with an alkali activator, including “geopolymers” [130]. The hydration system of AAMs can form N-A-S-H gel and C-A-S-H gel depending on the calcium oxide content [130]. In the process of geopolymerization, heavy metals not only can be physically encapsulated in the structure of geopolymers’ matrix with polycondensation reaction, but also can chemically bond with aluminate tetrahedral units or substitute the Al^3+^ position of geopolymers to be immobilized [131]. In some condition, geopolymers can be modified to a rough and irregular surface that is beneficial to the absorption of heavy metals [132]. Compared to OPC, AAMs are low in carbon and energy and have stronger performance characteristics such as enhanced temperature and chemical resistance.

#### 2.3.2. Thermal and Physical Process

Thermal processes that use heat to process waste include thermal desorption, incineration, vitrification, and thermoplastic polymer processes [14,92]. During thermal destructive processes, some amounts of metal are chemically incorporated into the glass structure in the form of oxides or other substances, and other metal components are separated and recycled for reuse [92]. Thermal-chemical approaches like Cement-lock^®^, Novosol^®^, and HPSS^®^ technology are widely applied (Table 4) [113,114,133]. The thermoplastic polymer process uses the property of thermoplastic polymers to become flowing when heated and solid when cooled to encapsulate contaminants. Non-thermal encapsulation is to physically separate the wastes from the environment, such as organic polymer microencapsulation processes and macroencapsulation/containerization [134,135].

### 2.4. Merits and Demerits

Ex situ remediation normally has high remediation efficiency and the whole process is under control. It can completely remove contaminants in sediment with quick results, but it requires a large investment and covers a wide area. Newly exposed post dredging residuals may lead to persistent exposure and risk to biota. As for dredged sediment, most remediation methods are basically increasing the mobilization or immobilization of metals to reduce toxicity and bioavailability.

Sediment washing is the most typical technique of ex situ technology, which is much simpler and more efficient than others. This technique is more suitable for coarse particles that heavy metals are weakly associated with due to their low area to volume ratio and negligible surface charge [10]. However, dredged sediment is usually fine grained and strongly adsorbed to heavy metals, making it difficult to wash, which is an important limitation of the washing technique. The heavy metals in sediment are eventually transferred with the washing agents, so the contaminant containing solution should be treated with caution.

EKR is easy to operate and cost-effective. Since the applied electric field can easily reach deeply buried contaminants, EKR can be highly efficient even in soils/sediments with low permeability [136]. This is something no other technology can achieve. Thus, compared with gravels and sands, EKR is most suitable for low permeability porous substrates (e.g., clays and silts). However, the side effects and energy consumption of EKR are important limiting factors. If appropriate electrodes, electrolytes, voltage gradient, or furthermore combining with other techniques (such as PRB) were utilized, the efficiency and economics of EKR could be improved. Overall, EKR is an innovation that is almost in the experimental stage and considerable field-scale research is expected.

The application of floatation to the remediation of heavy metals in sediments is seldom studied at present. Floatation is suitable for treating fine-grained matrices, particularly the dredged sediment (best separation in the particle range 20–50 µm) that most metals exist in as sulfides [59]. However, particle size of metal-bearing sediment/soil <10 µm and >200–300 µm are less effective in conventional floatation processes due to the mechanical entrainment of the fine hydrophilic particles and incapability of carrying coarse and heavy particles for bubbles [137]. Moreover, the extractability of this method is lower than that of other remediation techniques [10]. In addition, microwave and ultrasonic-assisted extraction has the advantages of being labor-less, time-saving, and environmentally friendly. However, considerable studies are limited to the experimental size, and once they are scaled up and applied to industry, the excellent properties will be overwhelmed. The scaling complexity of microwave-assisted extraction lies in the low predictive capacity of theoretical modeling of an empty microwave cavity, the changeable of dielectric constant for a given compound, low penetration depth of microwaves, and the particular raw material [138]. Ultrasonic-assisted extraction is a highly nonlinear process that is also difficult to efficiently transfer from a small to large scale operation [138].

Additionally, chemical agents related to the above techniques may have some drawbacks on sediment. For example, HCl significantly dissolved carbonate in calcrete soil and had a significant negative effect on soil microbial and enzyme activities, while EDTA leaching increased the pH value of soil but had little effect on soil microbial and enzyme activities [139,140]. By contrast, biosurfactants are promising that the high-concentration HS washing (2000 mg/L) combined with zeolite stabilization effectively activated the biological function of the sediment [27]. Thus, the environmental protection and high removal rate of the chemical agents are the focus of future research.

Biological treatment is a low-cost, ecological and sustainable reclamation strategy. Phytoremediation can achieve good results but the pre-treatment will increase the volume of sediment substrate by about 20–30%. This method generally requires long treat time and the remediation effect is not very stable. On the laboratorial scale, bioleaching can achieve high remediation efficiency under the best operation conditions, but for large-scale applications, efficiency may be lost due to inhomogeneity and parameter fluctuation or gradient [87].

Cement-based S/S remediation technology for pollutants has been extensively used during the last few decades. OPC is a simple and cost-effective solidifying material but it has some defects of high greenhouse gases emissions, high porosity, and high pollutants leaching levels [141]. In recent years, green and low-carbon cements (including blended OPC, CAS, MC, and AAMs) have increased by substituting clinker with supplementary cementing material, which have better sustainability and durability that can be used for a wider range of waste disposal. Beyond that, limestone calcined clay cement is considered to be a sustainable S/S binder with better environmental protection and durability, but further research is expected to understand its potential to stabilize pollutants [141].

## 3. Resource Utilization

The treatment of dredged sediment emphasizes the principles of harmless reduction and resource utilization. As for traditional landfills, composting, and other sediment disposal processes, the moisture content of sediment should be frequently reduced to less than 60%. However, the new resourced approaches put forward higher requirements for sediment moisture content and geotechnical properties. At present, resource-oriented utilization is classified as ecosystem restoration, construction materials, and agricultural activities.

### 3.1. Ecosystem Restoration

Dredged sediment is beneficial when used in coastal/wetlands restoration and marsh creation projects [18,19,142,143,144,145]. In these processes, sediments provide substrates that nourish habitats and promote the recovery of coastal flora and fauna [146]. On one hand, sediment can be mechanically or hydraulically dredged and transported by pipelines and distributed in open water areas to replenish nutrients in the marshes [18,19]. On the other hand, the de-watered and solid dredged material can be settled to the target elevation, creating new land [19]. Normally, the raw sediments do not have adequate shear strength and bearing capacity to withstand the overburden stress caused by the superstructure, so they need to be solidified/stabilized before land creation [20]. Representative examples for restoration efforts are in coastal Louisiana (USA), Mississippi River Delta (USA), Prince Edward Island (Canada), the Yangtze River Delta (China), and so on [18,19,142,143,144,145].

### 3.2. Construction Materials

**Figure 2:**
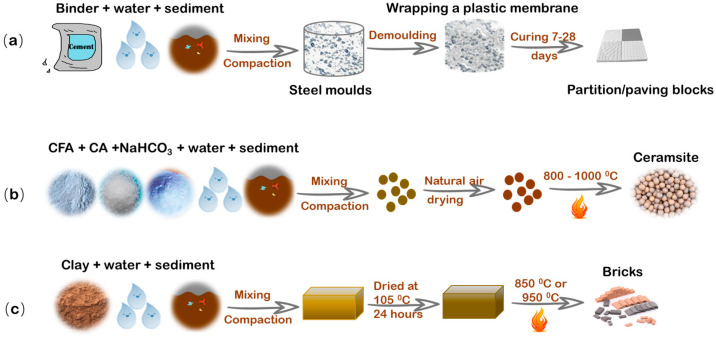
Possible processing stages of partition/paving blocks (**a**) ceramsite (**b**) and bricks (**c**) (This picture is reproduced according to [93,95,148,151]).

Based on the properties of the sediment itself and S/S technology, dredged sediment can be recycled as fill materials, partition blocks, paving blocks, ceramsite, supplementary cementitious materials, ready-mixed concrete, and foamed concrete (Figure 2). For example, Wang et al. [93] found that sediments with 5 wt% binder (OPC only or with incinerated sewage sludge ash or ground granulated blast-furnace slag) could fulfil strength requirements of fill materials, and 20–30% binder addition with dry-mixed and pressed method effectively densified the porous structure so that the strength fulfilled the requirements of partition blocks and paving blocks. Another pilot scale S/S project recycled contaminated marine sediments as filling materials by addition of lime, organoclay, and activated carbon [94]. Due to the high content of SiO_2_ (35–71%) and Al_2_O_3_ (4.1–18%), sediment can possibly be utilized for producing qualified ceramsite. Wang et al. [147] developed a novel ceramsite for water treatment using dredged river sediment, zeolite, and bentonite. The ceramsite meets the standard of lightweight ceramsite (bulk density was 0.89 g/cm^3^) with a specific surface area of 52 m^2^/g and a porosity of 54%. Cai et al. [148] successfully transformed the dredged sediment into an efficient water-absorbing ceramsite by mixing coal flyash, and its specific surface area and average pore size were 10 and six times higher than the commercial ceramsites, respectively. Likewise, the component of SiO_2_ and Al_2_O_3_ can also be used as a sand or clay replacement as a pozzolanic supplementary cementitious material (partial substitution of cement) in fired bricks and tiles [149,150]. Harbor dredged sediment were once used to fire clay bricks and their mechanical properties increased by 33% when sediments were added with 20 wt% and fired at 850 °C [151]. At the replacement level of 10–30% river sediment, 1–2% sewage sludge, and 1% wheat straw for shale, bricks with low thermal conductivity, light weight, and high strength were successfully manufactured in line with local standards [152]. Furthermore, as early as 1903, ready-mixed concrete was first produced in Germany, in which dredged sediments were applied as a fine or coarse aggregate in construction production. Research has shown that ready-mixed concrete made from 50% untreated- and 100% treated (desalination)-composite-marine dredged material instead of silica sand can meet the minimum requirement for C25/30 class [153]. Concrete C30/37 has been successfully developed by replacing admixture with 20% marine sediments and the total chloride was consistent with the required standards [154]. The research on foamed concrete is relatively limited. Yang et al. [155] designed an eco-friendly foamed concrete that mixed dredged sediment, cement, foam, and silica fume without heating and pressure, which is prospective.

### 3.3. Agriculture Activities

Dredged sediments contain a variety of organic matter (such as lignin oligomers, humic acids, chlorophylls, and carbohydrates) that can amend with soil to improve health [156,157], so there are many successful agricultural applications. For example, the dredged sediment from the Grand Canal (Hangzhou, China) was land used through pakchoi (*Brassica chinensis* L.) germination tests [158]. The results showed that dredged sediment utilization promoted plant growth when application rate is lower than 540 t/ha and applying up to a 15 cm thick layer of sediment for agricultural purposes is feasible. Tozzi et al. [159] applied remediated marine sediment as a growing medium for lettuce production; results showed that the plants were rich in minerals, organic acids, and antioxidant, but no symptoms of phytotoxicity were found. Martínez-Nicolás et al. [160] used port sediment as an agricultural medium for pomegranate cultivation, confirming that transfer of pollutants is limited and dredged sediments could be applied for agricultural activities. However, their applications were restricted due to the common pollutants (heavy metals and polycyclic aromatic hydrocarbons). Dredged marine sediments usually contain a high salt content that inhibits water absorption and plant growth [31]. If they are not treated properly, sediment application would hinder nutrient uptake by plants and increase the risks of heavy metals and organic pollutants [161]. Therefore, the maximum limits of heavy metal concentration for agricultural soils have been widely reported in general countries or regions. The imposed maximum concentrations, a proposal for a revision on the EU directive, and screening values from US and China in agricultural soils are exhibited in Table 5 [162,163,164,165,166,167,168,169,170].

## 4. Summary and Prospects

As a whole, the current work reviews the latest research to physical-chemical approaches and biotechnologies for treating metal-contaminated sediments. Generally, physical-chemical approaches are considered to be more effective and biological strategies are more environmentally friendly. However, the former are more expensive and the latter are time-consuming and have unstable efficiency. The adoption of remediation techniques is usually determined by sediment characteristics (such as the sediment texture, metal load, and species), function of the water body, financial and human costs, and many other factors. Furthermore, the environmental impact of these technologies should also be continuously monitored and analyzed.

Recycling dredged sediment for construction and building materials can be achieved through cement-based S/S technology. The main challenge is the heterogeneity of sediments coupled with various types of pollution (e.g., heavy metals), which requires various pre-treatment methods that adds considerable chemical/energy cost and treatment time. Repurposing sediment for ecosystem restoration, construction materials, and agricultural activities offers profitable alternatives to sediment as a waste disposal option, which are in line with policies around the world that support sediment as a resource. Moreover, the development of multi-way resource utilization for dredged sediment is conducive to environmental sustainability. In the future, it is hoped that polluted sediment can be comprehensively purified, and truly sustainable management of dredged sediment will be achieved.

## Figures and Tables

**Table 1:** Sediment washing for heavy metal contaminated sediment in recent years.

Sediment	Washing Agents	Heavy Metals	Findings	Reference
Göta Älv riverbed, Sweden	Fenton agent	Cd, Cu, Ni, Pb and Zn	The highest removal efficiency was 56% (Cd), 45% (Cu) and 40% (Zn).	[29]
Dongting Lake, China	HS	Cd	The removal efficiency ranged from 11% to 26%.	[27]
The marina Bjorlanda Kile, Sweden	EDDS, HPC, HA, iron colloids, ultra-pure Milli-Q water, “Soap” and NaCl	Cu and Zn	The highest removal efficiency of Zn (39%) was in HPC leached samples and Cu (33%) in EDDS leached samples.	[30]
A industrialized harbor, Korea	HCl, HNO_3_ and H_2_SO_4_	Zn, Cu and Cd	The HCl was verified as the most effective acid solutions, followed by HNO_3_ and H_2_SO_4_.	[31]
Dongting Lake and Qili Mountain, China	HS from the peanut straw, sesame straw, corn straw and deciduous leaves	Cd and Ni	HS from the sesame straw removed more than 74% of Cd and 42% of Ni from sediment.	[32]
Fuxing Island, China	Tea saponin, saponin and sophorolipid	Cu, Cd, Cr and Pb	Sophorolipid was the most effective ones with the removal efficiency of 39% (Cu) and 70% (Cd).	[33]
Huangpu River, China	Biosurfactant rhamnolipid	Cu, Cd, Pb and Cr	80%, 87%, 64% and 48% of Cu, Cd, Pb and Cr were removed by 0.8% rhamnolipid after 12 h at pH 7.0.	[26]
Shenzhen River, China	HCl, HNO_3_, CaCl_2_, Ca(NO_3_)_2_, EDTA, EDDS, H_2_O_2_, Na_2_S_2_O_8_, CA and Fenton agent	Cr, Cu, Pb and Zn	Inorganic acids were the most effective ones with the removal efficiency of 16–53% (Cr), 74–88% (Cu), 71–93% (Pb) and 83–87% (Zn).	[34]
Yingkou Port, China	CA, malic acid, tartaric acid, and oxalic acid	Cu, Cd, and Pb	The average removal rates were 85%, 73%, 56% and 35% for CA, malic acid, tartaric acid and oxalic acid, respectively.	[35]
Augusta Bay, Italy	EDTA, CA and AA	Ni, Cu, Zn, Cr, and Hg	The removal efficiencies (CA) ranged from 78 to 82% for Ni, Cu, Zn and Cr.	[36]
One polluted river, China	CA, AA, malic acid and succinic acid	Pb, Cd, Zn and Cu	AA showed high extraction capacity while CA showed no obvious leaching effect on Pb and Cu.	[37]

**Table 2:** Ex situ EKR for heavy metal contaminated sediment in recent years.

Sediment	Type	Techniques	Heavy Metals	Findings	Reference
The Caspian Sea, Iran	Sediment	EKR with microbial fuel cell	Cr	Anode and cathode achieved up to 73% and 54% removal efficiency of Cr^6+^.	[50]
A mine pond, Turkey	Sediment	EKR	As, Al, Fe and Mn	Al and Mn were able to be removed, but the removal of Fe and As was negligible.	[51]
The Augusta Bay, Italy	Sediment	EKR with both MGDA and Tween 80 as processing agents	Hg	The best Hg-removal (~71% in 400 h) was achieved.	[52]
The Fereydounkenar beach, Iran	Sediment	EKR with microbial fuel cell	Cr	The removal efficiency was 68%.	[53]
Wuhan East Lake, China	Sediment	EKR with KMnO_4_ as oxidant, enhanced by CA	Cd	The removal rate of total Cr reached 61%.	[54]
A harbor in Tancarville, France	Sediment	EKR enhanced by CA	As, Cr, Cd, Zn, Ni, Cu and Pb	The greatest removal efficiency was more than 50% for Cr.	[55]
Veliki Bački canal, Serbia	Sediment	Hexagonal two dimensional electrokinetic system with solar panels	Ni	Total efficiency of removing Ni is more than 62%. Cumulative energy consumption is 0.74 kWh/kg.	[56]
Veliki Backa canal, Serbia	Sediment	EKR with solar cells	Ni, Cd and Zn	3%Ni, 82%Cd and 58%Zn were removed from the anode region.	[57]
A harbor in Tancarville, France	Sediment	EKR enhanced by CA and biosurfactants (rhamnolipids and saponin)	Cd, Cr, Cu, Pb, Zn	Removal levels (4.4–16%) were not as high as expected.	[58]

**Table 3:** Chemical extraction for heavy metal contaminated sediment in recent years.

Sediment	Techniques	Chemical Agents	Heavy Metals	Findings	Reference
Guadalhorce river, Málaga, Spain	Microwave-assisted sequential extraction	AA, NH_3_OHCl, H_2_O_2_	Cu, Ni, Cr, Pb and Cd	Detection limits were 1–18 ng/L. The recovery was from 82% to 103%.	[66]
Hussain Sagar lake, India	Microwave-assisted extraction	EDTA, C₁₆H₃₆BrN and HF	Cr	The detection limit was 5.0 mg/kg. The recovery was 96–99%.	[72]
Lami coastal, Fiji	Microwave-assisted extraction	aqua regia	Al, Cr, Cu, Fe, Mn, Ni, Pb and Zn	Recovery values were above 82%.	[73]
Mokolo River catchment, South Africa	Microwave-assisted extraction	HCl, HF and Na_2_CO_3_	Cr	The recovery was 94.9–105%.	[74]
The river Hornád, Slovakia; a fishpond, Gödöllő	Microwave-assisted extraction	AA, HNO_3_/H_2_O_2_	Zn, Cd, Pb, Ni, Cr, Cu	The efficiency does not reach that of the (3 + 1)-step BCR procedure.	[75]
BCR-701 sediment (CRM)	ultrasound-assisted sequential extraction	HNO_3_/H_2_O_2_	Cd, Cr, Cu, Ni, Pb and Zn	77% of element content can be extracted.	[76]
Msunduzi River, South Africa	Ultrasonic and microwave assisted extraction	HNO_3_	Cr, Cu, Cd, Ni, Pb and Zn	The recoveries were 80–98% with the microwave-assisted and 79–103% with ultrasonic-assisted, respectively.	[77]
The hydrographic basin, Rio Doce	Ultrasound-assisted extraction	HCl, HNO_3_, H_2_O_2_ and HF	Cr, Cu, Zn, Cd, and Pb	Recoveries ranged from 80.1 to 93.7% for CRM and from 89 to 107% for spike tests.	[78]
Estuarine sediment reference materials	ILs mediated and ultrasound-assisted extraction	NaHCO_3_, HCl, HNO_3_, HF and BF_4_	Cd, Cr, Cu, Ni, Pb and Zn	The recovery was 81–102% with RSD <9.5%.	[79]
Bahía Blanca Estuary, Argentina	ILs mediated and ultrasound-assisted extraction	EDTA, NaHCO_3_, BF_4_	Cd, Cr, Cu, Ni, Pb and Zn	The extraction time was reduced to 7.0 min.	[65]

**Table 4:** S/S technology for heavy metal contaminated sediment in recent years.

Location	Binding Materials	Heavy Metals	Main Findings	Reference
Fuxing Island Canal, China	OPC or CSA	Cu and Cd	The solidification effect of OPC on Cu was better than that on Cd, even in acidic conditions.	[96]
Fuxing Island Canal, China	Cement and TMT	Cd and Pb	The leaching concentrations met the requirement of in situ resource recycling standard after 28 days.	[97]
Marine, Hong Kong, China	Lime and ISSA	Zn and Cu	Very low leaching concentrations of Zn (<0.1 mg/L) and Cu (≤0.05 mg/L) were detected after 28 days.	[98]
Lake Nanhu, China	GGBS and MgO	Zn	Leachability of Zn was below the regulatory limit of the Chinese standard method (GB 5085.3-2007).	[99]
A commercial harbor, Ireland	OPC and GGBS	Al, Cr, Mn, Fe, Ni, Cu, Zn, Cd and Pb	Immobilization efficiencies were more than 98% for metals over 100 days.	[100]
Taihu Lake, China	OPC	Cr, Cu, Zn, Pb, Ni, and Hg	The S/S sediment complied with the acceptance criteria (GB5085.3-2007) in terms of metal release.	[101]
Marine, Hong Kong, China	Lime and ISSA	Cu, Zn and Pb	Negligible toxic metals were leached and only very small amounts of Cu, and Pb could be detected.	[102]
Great Bačka Canal, Serbia	Kaolinite, quicklime and OPC	Cr, Ni, Cu, Cd, Zn, Pb	Leaching concentrations of Cr, Cu, Zn, Cd and Pb didn’t exceed prescribed values after 7 years.	[103]
Sembrong River, Malaysia	Cement and rice husk ash	Cu	The retention capacity of Cu spiked sediment was from 92% to 99%.	[104]
Marine, Marpiccolo, Italy	OPC, lime, activated carbon and organoclay	Co, Cr, Ni, Pb, Cu and Zn	The leaching of each metal has to be lower than limits imposed by legislation.	[105]
Kwun Tong Typhoon Shelter, China	OPC, industrial by-products (i.e., PFA, ISSA, GGBS, and CCR) and CO_2_	Zn, Cu, Pb and Cr	Leaching concentrations of heavy metals were under the universal treatment standard in Hong Kong.	[106]
Kwun Tong Typhoon Shelter, China	OPC, MOC and CO_2_	Zn, Cu, Pb and Cr	Leaching concentrations of heavy metals were under the universal treatment standard in Hong Kong.	[95]
Tangdao bay, China	epoxy resin and alkali-activated GGBS	Fe, Cd and Cr	The maximum content of heavy metals were much lower than the respective contamination limits set by US EPA.	[107]
Sembrong River, Malaysia	Cement and rice husk ash	Pb	Leaching concentration was below the limit of 5 mg/L after 28 days of curing.	[108]
Hong-xing Lake, China	Cement, fly ash and slag	Cu, Pb, Cd, Zn, Cr and Ni	Leaching concentrations were all lower than the Chinese standard method (GB5085.3-2007).	[109]
The Pearl River Delta, China	Cement, lime and bentonite	Cd, Cu, Cr, Ni, Pb and Zn	The leached concentration of Cd, Cr, Cu, Ni, and Zn decreased by more than 80%.	[110]
New Jersey harbor area, New York	Cement-lock^®^ technology	Cd, Cr, Cu, Pb, and Hg	The leachability of metals is several orders of magnitude below regulatory limits.	[111]
Nord-Pas-de-Calais region, France	Novosol^®^ porcess	Cd, Cr, Cu, Pb and Zn	Leaching concentrations were largely within regulated limits given by the Commission of European communities.	[112]
Xiawan port, China	Novosol^®^ porcess	Cd, Zn and Pb	Leaching concentrations were all lower than the Chinese standard method (GB5085.3-2007).	[113]
Mincio river, Italy	HPSS^®^ technology	Hg	Volatile pollutants are removed.	[114]

**Table 5:** Limit values for heavy metal concentration in agricultural soils.

Heavy Metal	Limit Concentrations (mg/kg Dry Weight)				
	EU, Current Value ^a^ (6 ≤ pH < 7)	EU, Proposal ^a^ (6 ≤ pH < 7)	US, Screening Value ^b^ for Plant	China, Screening Value ^c^ (6.5 < pH ≤ 7.5)	China, Control Value ^c^ (6.5 < pH ≤ 7.5)
Cd	1–3	1	32	0.3	3.0
Hg	1–1.5	0.5	NA	2.4	4.0
As	NA	NA	18	30	120
Pb	50–300	70	120	120	700
Cr	NA	75	NA	200	1000
Cu	50–140	50	80	100	NA
Ni	30–75	50	38	100	NA
Zn	150–300	150	160	250	NA

NA = not available. ^a^ Vareda, J.P., Valente, A.J.M. and Duraes L. (2019). ^b^ United States Environmental Protection Agency (2005–2008). ^c^ Ministry of Ecology and Environment of the Peoples’s Republic of China (2018).

## Data Availability

Not applicable.

## Abbreviations

Fe-Mn	iron-manganese
EKR	electrokinetic remediation
EDDS	ethylene diamine disuccinate
AA	acetic acid
HS	humic substances
HA	humic acid
PRB	a permeable reactive barrier
Tween 80	polyoxyethylene (20) sorbitan monooleate
BCR	The Community Bureau of Reference
BF_4_	bmim
OPC	ordinary Portland cement
MC	magnesia-based cements
ISSA	incinerated sewage sludge ash
PFA	class-F pulverized fly ash
MOC	magnesium oxide cement
CFA	coal fly ash
Co	cobalt
AAMs	alkali-activated materials
EDDS	ethylene diamine disuccinate
CA	citric acid
HPC	hydroxypropyl cellulose
“Soap”	saponified tall oil
MGDA	methylglycinediacetic acid
ILs	ionic liquids
RSD	relative standard deviation
CRM	certified reference material
S/S	solidification/stabilization
CSA	calcium sulfoaluminate cement
TMT	rimercapto-s-triazine trisodium salt
GGBS	ground granulated blastfurnace slag
CCR	calcium carbide residue
GB5085.3-2007	Chinese standard method: Identification standards for hazardous wastes—Identification for extraction toxicity
